# The journey of maternal platelets through the utero-placental circulation: routes, interactions, and physiological implications

**DOI:** 10.1093/molehr/gaag023

**Published:** 2026-04-25

**Authors:** Désirée Forstner, Martin Gauster, Berthold Huppertz, Freya Lyssy

**Affiliations:** Division of Cell Biology, Histology and Embryology, Gottfried Schatz Research Center, Medical University of Graz, Graz, Austria; Division of Cell Biology, Histology and Embryology, Gottfried Schatz Research Center, Medical University of Graz, Graz, Austria; Division of Cell Biology, Histology and Embryology, Gottfried Schatz Research Center, Medical University of Graz, Graz, Austria; Division of Cell Biology, Histology and Embryology, Gottfried Schatz Research Center, Medical University of Graz, Graz, Austria

**Keywords:** placenta, utero-placental circulation, platelets, trophoblast, maternal hemodynamics

## Abstract

Maternal platelets fulfil an exceptional and multifaceted plethora of roles during pregnancy, extending far beyond their traditional function in hemostasis. Increasing evidence suggests that platelets participate in placental development, immune modulation, and vascular adaptation at the maternal–fetal interface. This review traces the journey of maternal platelets from a possible adaptation of megakaryopoiesis in the maternal bone marrow to their first encounter with trophoblast cells during early placentation, continuing their way into the intervillous space and eventually leaving the utero-placental circulation, returning to maternal peripheral blood. Important hemodynamic changes during pregnancy, including a reduction in platelet count and an augmented uterine blood flow, will be highlighted. Initial contact of maternal platelets with invading extravillous trophoblasts at the sites of plugging of invaded spiral arteries is underscored, with further discussion of the influence on trophoblast behavior, vascular remodeling, and local inflammatory signaling. Furthermore, following the routes by which platelets access the intervillous space and interact with the syncytiotrophoblast, the dynamic exchange of soluble mediators, surface receptors, and extracellular vesicles is explored. The bidirectional nature of platelet–trophoblast communication is discussed, as are its potential implications for placental function in both physiological and pathological pregnancies. Finally, the possibility is addressed that platelets, following placental interaction, re-enter the maternal vasculature with altered phenotypic or functional properties. This review emphasizes the importance of maternal platelets as active regulators of placental biology and pregnancy outcome by synthesizing current insights.

## Maternal platelets: from bone marrow to uterus

Human platelets are small, discoid-shaped cells present in circulating blood. On average, they measure a mere 2–3 µm in diameter and 0.5 µm in thickness (Michelson, 2013). Platelets are well-known for their function in blood coagulation and wound healing; however, these cells may offer and support way more pathways than originally assumed (Maouia *et al.*, 2020).

Platelets are produced by extension of branches of large polyploid cells, so-called megakaryocytes, into the sinusoids of the bone marrow. Megakaryocytes originate from hematopoietic stem cells (HSCs) (Machlus and Italiano, 2013; Boscher *et al.*, 2020). During their maturation process, megakaryocytes increase their size and are filled with platelet-specific granules. They expand their cytoplasmic content of cytoskeletal proteins and develop a highly tortuous invaginated membrane system (Machlus and Italiano, 2013).

**Figure 1.**
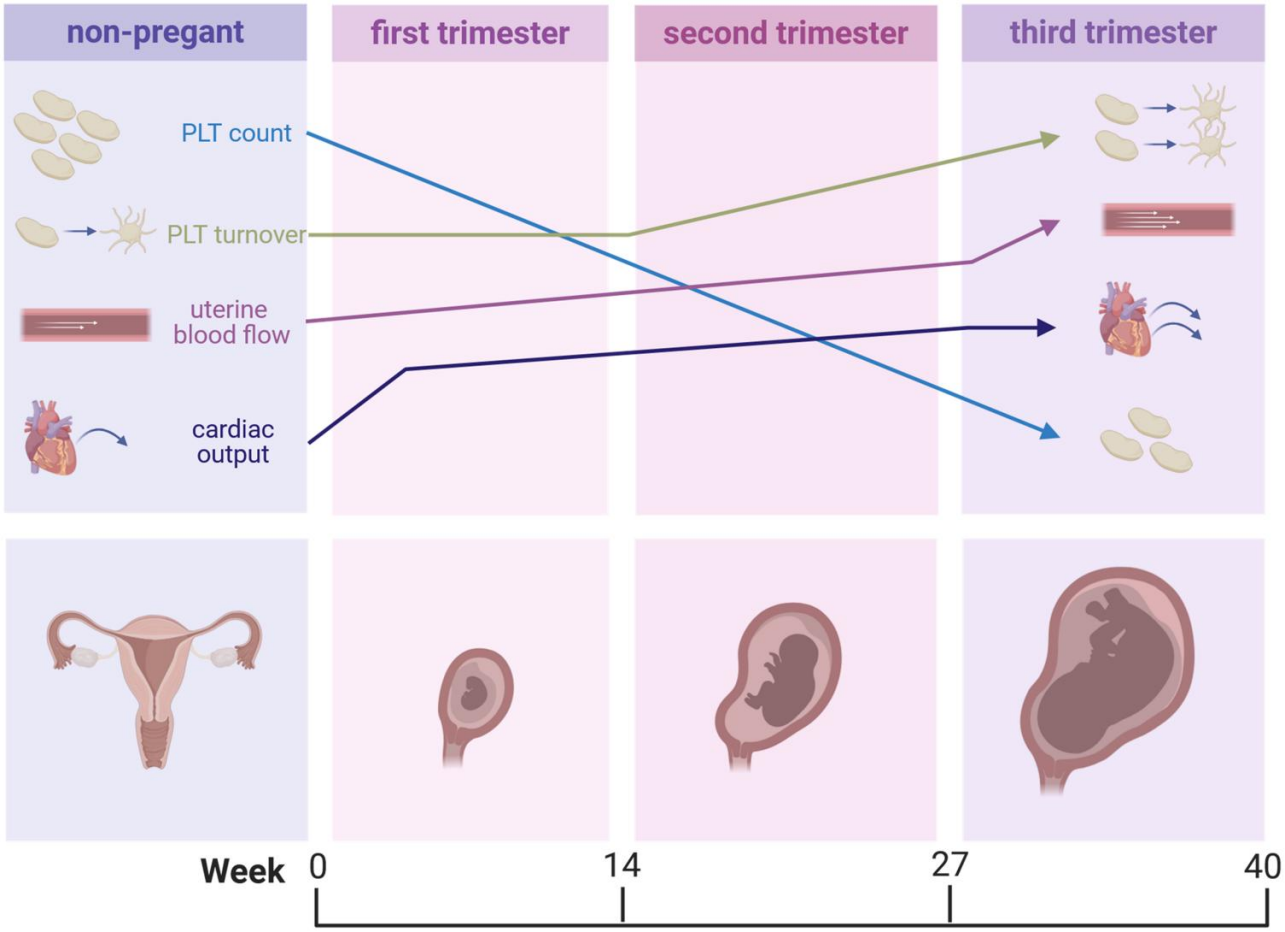
**Maternal hemodynamics are changing during pregnancy**. Compared to a non-pregnant stage, several hemodynamic factors in the mother change during pregnancy. While platelet (PLT) count gradually decreases with ongoing pregnancy, PLT turnover starts to increase only from the second trimester onwards. To adapt adequately to fetal growth and demands, uterine blood flow increases with ongoing gestation, with the largest rise toward the third trimester. Maternal cardiac output increases early, rises through mid-pregnancy and plateaus towards term. Created in BioRender. Gauster, M. (2026), https://BioRender.com/jmomx3i.

Pregnancy is associated with a progressive increase in platelet turnover characterized by shortened platelet lifespan, increased peripheral consumption, and compensatory upregulation of thrombopoiesis, changes that become most pronounced in the third trimester (McCrae, 2010; Fogerty and Dzik, 2021). In healthy non-pregnant women, a platelet count between 1.5 and 4.0 × 10^5^ platelets per µl whole blood is considered normal. Interestingly, the maternal platelet count undergoes a gradual decline from the first to the second to the third trimester, with a total decrease of approximately 10% at term in healthy pregnancies (Fig. 1 and Table 1) (Michelson, 2013; Moser *et al.*, 2019). Recent studies have suggested that during gestation, several steps of megakaryopoiesis as well as thrombopoiesis can be altered, which can ultimately lead to modified platelet content and function (Dupuis *et al.*, 2019; Szklanna *et al.*, 2019).

**Table 1.** Cardiovascular and platelet adaptations during pregnancy.

	First trimester	Second trimester	Third trimester
PLT count	↓	↓↓	↓↓↓
MPV	↑	↑	↑
PDW	↓	↓	↓
IPF	↔/↑	↑	↑↑
PLT turnover	↔/↑	↑	↑↑
PLT lifespan	↔/↓	↓	↓↓
Uterine blood flow	↑↑	↑↑↑	↑↑↑
Cardiac output	↑	↑↑	↑↑
Maternal heart rate	↑	↑↑	↑↑↑
Maternal BP	↓↓	↓↓↓	↓↓↓
Spiral artery BP	↓↓	↓↓↓	↓↓↓
Uterine vein BP	↓	↓↓	↓↓

Arrows indicate an increase or decrease in the according trimester compared to the non-pregnant state; BP, blood pressure; IPF, immature platelet fraction; MPV, mean platelet volume; PDW, platelet distribution width; PLT, platelet.

It has previously been suggested that platelets can be differently ‘educated’ during physiological stressful conditions such as pregnancy, leading to diverse content of their platelet-released factors. Such ‘educating’ factors include proteins that are exclusively expressed during pregnancy, like human placental lactogen or pregnancy-specific glycoproteins (Szklanna *et al.*, 2019). However, these factors might also include extracellular vesicles (EVs), which are shown to be functionally and phenotypically different during pregnancy, especially in women diagnosed with preeclampsia (Kohli *et al.*, 2016).

Even before platelets may get primed by the pregnant environment, studies have suggested that megakaryopoiesis is already adapted to pregnancy. Healthy pregnant women show elevated thrombopoietin (TPO) levels compared to healthy non-pregnant women (Frölich *et al.*, 1998). TPO is the key cytokine driving platelet production; hence, higher TPO levels would suggest an elevated platelet count (Kuter, 2022). However, during pregnancy, a controversial paradigm seems to exist with higher TPO levels and a decreased platelet count. It is currently assumed that, in addition to the dilution of platelets resulting from plasma volume expansion, a number of other physiological changes occurring during pregnancy, including accelerated platelet sequestration and consumption in the utero-placental circulation, account for lower platelet counts (Reese *et al.*, 2017, 2019).

Moreover, studies have reported an increase in immature platelet fraction (IPF) values from week 20 to week 40 of gestation (Ratsch *et al.*, 2017). The IPF is defined as the percentage of immature platelets compared to the total number of platelets in peripheral blood, which can be used to reflect the activity of the bone marrow to produce new platelets (Benlachgar *et al.*, 2020). This again would suggest an elevated stimulation of megakaryopoiesis, especially during later weeks of gestation, to possibly compensate for subclinical consumption (Ratsch *et al.*, 2017). Immature platelets show features of higher functional activity compared to ‘older’ platelets, e.g. larger size and higher RNA content, as well as higher levels of activation-related transcripts and proteins (e.g. receptors for thrombin), and greater responsiveness to agonists and higher surface activation markers (e.g. P-selectin) after stimulation (Bernlochner *et al.*, 2015; Gumiężna *et al.*, 2023). Although there were no significant changes in platelet distribution width (PDW), an increase in mean platelet volume (MPV) confirms the existence of younger platelets and therefore an enhanced platelet turnover in pregnancy (Table 1) (Han *et al.*, 2014).

All these facts align with the peculiarity that the maternal coagulation system seems to shift towards a pro-thrombotic and pro-aggregatory state during pregnancy (Forstner *et al.*, 2021). Although there are evolutionary reasons behind this to diminish possible risks for major bleedings during utero-placental vascular remodeling, parturition, and postpartum uterine involution, excessive platelet activation may also trigger inflammasome activation and eventually lead to potentially dangerous sterile inflammation in the mother (Brenner, 2004; Li and Huang, 2009; Moser *et al.*, 2019). In hypertensive disorders during pregnancy (e.g. preeclampsia, gestational hypertension), the above-mentioned changes in platelet characteristics are reported to be even more pronounced, and might therefore be at least partly responsible for the ongoing inflammation (Gardiner and Vatish, 2017; Moser *et al.*, 2019).

## Pregnancy induces changes of hemodynamic factors

### Adaptations of the cardiovascular system enable maternal–fetal perfusion

Overall, maternal cardiac output increases throughout pregnancy with the sharpest rise during the beginning of the first trimester and a continued increase into the second trimester (Fig. 1). Pregnancy is characterized by a systemic vasodilation and a substantial decrease in peripheral vascular resistance (Sanghavi and Rutherford, 2014). Besides extensive research considering the involvement of the placenta in the development of preeclampsia, pregnancy complications like gestational hypertension and preeclampsia clearly belong to the group of cardiovascular disorders (Melchiorre *et al.*, 2022). The hemodynamic balance between flow and resistance is the key variable to describe the different mechanisms of hypertension. Volume expansion is necessary to ensure a sufficient utero-placental perfusion, but also carries risks, as it is a stressor for the cardiovascular system (Gyselaers and Dreesen, 2025). To support the adaptation of the maternal cardiac system, the mother’s heart rate increases progressively, reaching a maximum increase of about 10–20 bpm in the third trimester. However, arterial blood pressure, including systolic as well as diastolic blood pressure, decreases sharply during the first trimester, reaching its minimum during the second trimester (Sanghavi and Rutherford, 2014). As many of these changes already occur early in pregnancy, it can be assumed that early placentation and maternal adaptations are highly important in avoiding any imbalance in the aforementioned cardiovascular as well as platelet characteristics (Table 1) and therefore in preventing poor pregnancy outcomes (Huppertz, 2024, 2026a).

### Uterine vascular remodeling supports utero-placental blood flow

**Figure 2.**
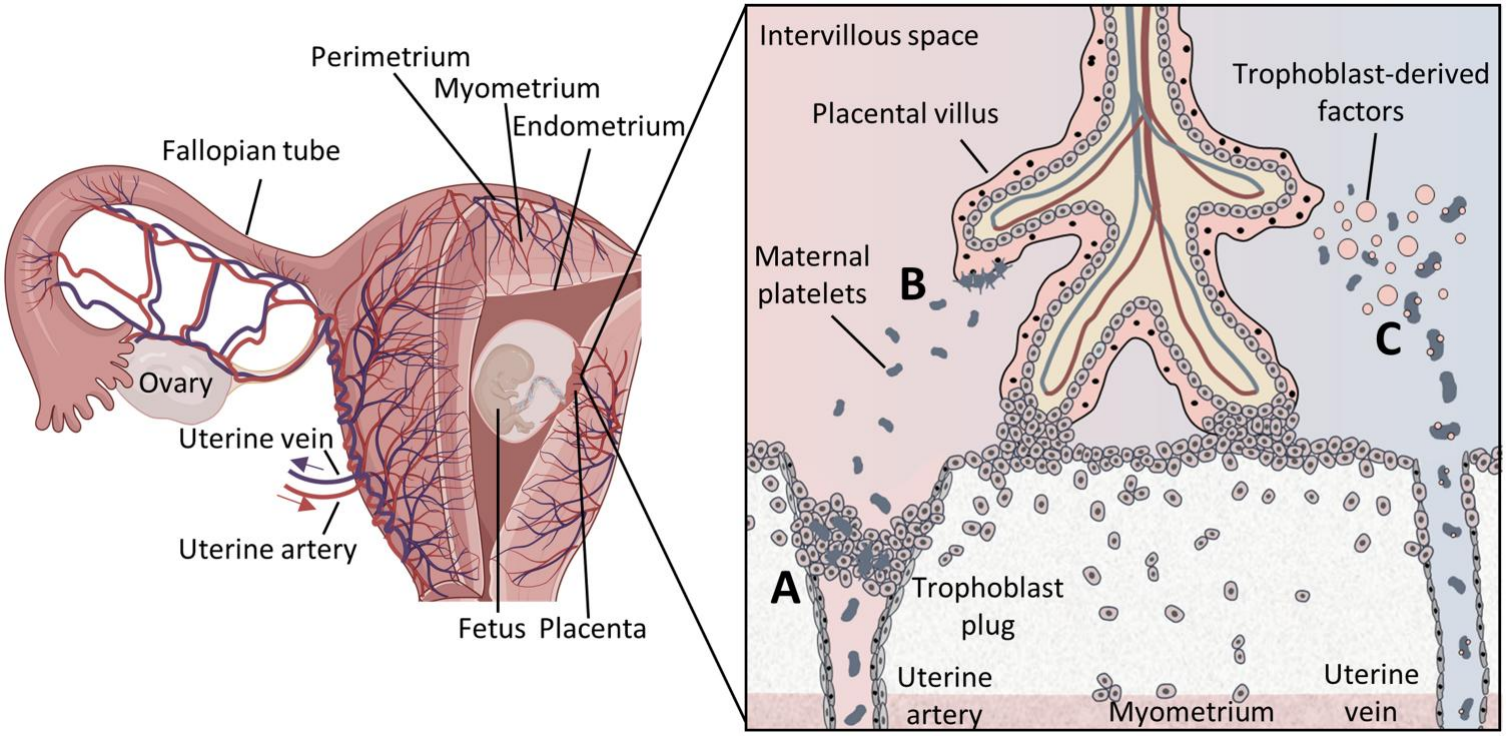
**Initial contact of maternal platelets with fetal trophoblasts**. The uterus is a highly vascularized organ, with the main blood supply coming from the internal iliac arteries. The continuing uterine arteries have several branches supplying the uterus as well as the cervix, vagina, fallopian tubes, and ovaries with blood. During pregnancy, remodeled spiral arteries in the functional layer of the decidua basalis are responsible for supplying the placenta with maternal blood. Before maternal blood flow commences toward the intervillous space, endoarterial trophoblasts form plugs within the lumen of the spiral arteries. However, platelets can percolate between the cells of the plugs at an early stage of pregnancy (**A**). Platelets can also adhere to the syncytiotrophoblast (**B**) or interact with trophoblast-derived factors (**C**). Partly created in BioRender. Gauster, M. (2026), https://BioRender.com/8wp1dwg.

Once synthesized by the bone marrow and released into the systemic circulation, platelets flow in arterial and venous blood without any organ specificity. To reach the uterus, they need to enter the common iliac artery, which branches into the internal iliac artery, finally leading to the uterine artery. From the uterine artery, several branches sprout off, which are responsible for supplying blood to not only the uterus, but also the cervix, vagina, fallopian tubes, and ovaries (Drenckhahn and Asan, 2004). From the uterine artery, the journey of platelets continues into the myometrium via the arcuate arteries, and to the endometrium via the radial arteries, finally entering spiral arteries within the basal and functional layers of the endometrium, where vessels eventually branch to form a dense subepithelial capillary plexus situated immediately beneath the uterine epithelium (Fig. 2) (De Ganzo Suárez *et al.*, 2024).

During placentation, a hemochorial maternal–fetal barrier is established, exposing maternal blood directly to the surface of the villous trophoblast. This creates a unique vascular compartment, the intervillous space, characterized by low-pressure, low velocity, high-volume flow, and complex shear gradients. It has been estimated that around 60–100 spiral arteries are responsible for supplying the term human placenta with maternal blood (Martin, 1965; Moore *et al.*, 2016). Uterine blood flow increases 10–15-fold during pregnancy, rising from approximately 50 ml/min in non-pregnant women to 500–800 ml/min at the end of pregnancy (Fig. 1 and Table 1) (Osol and Mandala, 2009; Hu and Zhang, 2021; Moore *et al.*, 2022).

Remodeling of uterine spiral arteries is an essential step to ensure the healthy continuance of pregnancy, as these arteries need to be adapted from small, high-resistance, vasoreactive vessels into large-caliber, low-resistance, non-vasoactive conduits to ensure the placenta receives a steady, low-pressure but high-volume supply of maternal blood (Osol and Mandala, 2009; Albrecht and Pepe, 2020). Trophoblast remodeling mediates loss of most of the vascular smooth muscle cells, resulting in a drop in arterial pressure at the distal ends of invaded spiral arteries from 60–80 mmHg to approximately 10–15 mmHg to allow gentle maternal perfusion of the placental villi and prevent shear-related damage (Table 1) (Burton *et al.*, 2009). This incremental transition from an endothelium-lined artery to a non-endothelialized conduit has important implications for platelet behavior. This is the first time platelets come into contact with fetal trophoblast; this time, the extravillous trophoblast. Later during their journey, platelets are exposed to the surface of the syncytiotrophoblast, locally produced fetal soluble mediators, and extracellular components, such as fibrin deposits within the intervillous space (Reese *et al.*, 2019).

## The uterine artery delivers platelets to their first contact with trophoblasts

During early human placentation, distal trophoblasts of evolving anchoring villi form trophoblast cell columns, which anchor the developing placenta to the highly differentiated uterine mucosa, called decidua. These cells differentiate into an invading phenotype, the extravillous trophoblast (EVT), which penetrates several luminal structures of the decidual stroma, including spiral arteries, veins, glands, and lymphatic vessels (Moser *et al.*, 2015; Guettler *et al.*, 2021; Huppertz, 2026b). A characteristic of the remodeling of spiral arteries is the formation of trophoblast plugs within these blood vessels to impede the entry of maternal blood cells into the early intervillous space (Weiss *et al.*, 2016). Only at the beginning of the second trimester, maternal blood flow into the intervillous space is fully granted (Roberts *et al.*, 2017).

Interestingly, it has been discussed that cells of the trophoblast plugs within the spiral arteries might already become vaguely cohesive, building capillary-shaped channels starting in the second half of the first trimester (Roberts *et al.*, 2017). Despite the negligible size of these emerging intercellular gaps, platelets, given their small diameter of 2–3 µm, may have the potential to enter these spaces before any other maternal blood cells. In fact, over 15 years ago, the question was first posed as to whether circulating blood cells might contribute to maternal tissue remodeling and embryo–maternal cross-talk during implantation processes (Fujiwara, 2009). Since then, studies have localized maternal platelets in trophoblast plugs, giving rise to further questions regarding a possible trophoblast–platelet interaction during early placental development (Fig. 2) (Sato *et al.*, 2005; Guettler *et al.*, 2021; Lyssy *et al.*, 2025b).

Although previous studies have shown that HLA-G, a protein strongly expressed by EVTs, does not affect trophoblast aggregation and adhesion, several components of matrix-type fibrinoid secreted by EVTs, consisting of laminins, collagen type IV, vitronectin, heparan sulfate, and cellular fibronectins, are potential substrates for platelet receptors that might trigger their activation (Kaufmann *et al.*, 1996; Kemp *et al.*, 2002; Guettler *et al.*, 2021; Gauster *et al.*, 2022). Furthermore, it cannot be neglected that platelets might get pushed into these intercellular gaps of trophoblast plugs due to maternal plasma flow towards the intervillous space, hence shear stress could also cause activation of platelets at these sites (Guettler *et al.*, 2021).

Upon activation, platelets release their cargo and cause possible downstream mechanisms to be activated in their surroundings (Koenen, 2016; Guettler *et al.*, 2022). In fact, it has been discussed that platelet-derived factors can influence the expression profile of invading trophoblasts, which might subsequently interfere with trophoblast invasion processes and spiral artery remodeling (Sato *et al.*, 2005; Lyssy *et al.*, 2025b). In a recent study, LAIR2-signaling was identified as a potential pathway influenced by platelet-derived factors (Lyssy *et al.*, 2025b). Notably, certain genes were upregulated in trophoblasts in response to platelet-derived factors that, in return showed to have an impact on platelet activation. Therefore, it has been suggested that there might be a vivid crosstalk between maternal platelets and extravillous trophoblasts, leading to a fine-tuned regulatory feedback mechanism at the early maternal–fetal interface (Lyssy *et al.*, 2025b).

In this context, it is necessary to mention that these platelet-derived factors include epidermal growth factor (EGF), vascular endothelial growth factor (VEGF), and platelet-derived growth factor (PDGF). All of these factors seem to enhance infiltration of a specific population of trophoblasts, so-called endoarterial EVTs, into the walls of spiral arteries (Moser and Huppertz, 2017; Huppertz, 2026b). Although it has been suggested that maternal platelets are not an essential component for human placentation, pregnancy-related pathologies, such as preeclampsia, are characterized by abnormalities in pro- and anticoagulant, and fibrinolytic pathways as well as changes in platelet morphology, numbers, and function (Sato *et al.*, 2010; Skeith *et al.*, 2020).

Hemochorial placentation is the most invasive type of placentation amongst eutherians, demanding the maternal immune system to quickly adapt to tolerate the semi-allogeneic fetus (Svensson-Arvelund *et al.*, 2014). Although recent single-cell RNA-sequencing studies have reported over 20 different types of immune cells to be involved in the immune cell population at the maternal–fetal interface in early pregnancy, platelets have largely been neglected due to their lack of a nucleus and their low RNA content (Vento-Tormo *et al.*, 2018; Guettler *et al.*, 2022).

However, platelets might act as local innate immune regulators by releasing a handful of immunomodulatory mediators (e.g. TGF-β, chemokines, and extracellular vesicles) (Semple *et al.*, 2011; Ribeiro *et al.*, 2019). This could influence the recruitment of decidual immune cells, including macrophages and uterine natural killer (dNK) cells, possibly promoting maternal immune tolerance (Schrottmaier *et al.*, 2020).

Activated platelets can interact with neutrophils, monocytes, and T-cells via mediators such as P-selectin, CD40 ligand, RANTES, and PF4, promoting a platelet–leukocyte interaction, neutrophil extracellular traps, and proinflammatory cytokines (e.g. IL-6, TNF-a), driving coagulation (Kohli and Blois, 2025). Besides the release of those mediators, platelets also express immune receptors, including glycoprotein VI, integrins (such as GPIIb/IIIa), or toll-like receptors, which need to be further investigated in the context of pregnancy (Kohli and Blois, 2025). Interestingly, it has also been shown that platelets interact with tissue-resident macrophages, e.g. with Kupffer cells from the liver, leading to an amplified phagocytosis (Mantovani and Garlanda, 2013). Whether or not platelets can also interact with macrophages from the placenta or uterine tissue remains to be elucidated. However, one might speculate about a possible interaction between macrophages and extravasated platelets in areas of vascular leakage, especially at sites of massive trophoblast invasion.

Important to mention here is the fact that hypertensive disorders during pregnancy are not considered purely cardiovascular but rather multisystemic disorders. In addition to endothelial dysfunction and hypertension, these disorders involve profound alterations in maternal immune regulation and placental development (Qin and Long, 2025; Huppertz, 2026a). Under pathological conditions, platelets may be responsible for a shift in T-cell differentiation towards more pro-inflammatory types (e.g. Th1, Th17). This could potentially disrupt immune tolerance, promoting a range of adverse pregnancy outcomes (Kohli and Blois, 2025).

## Entering of platelets into the intervillous space brings new hemostatic challenges

As platelets might be the first cells to enter the intervillous space at around 5 weeks of gestation, they are the first to interact with the surface of placental villi, which is constituted by the syncytiotrophoblast (STB) (Forstner *et al.*, 2020). Once the villous cytotrophoblast differentiates into the STB, it synthesizes important hormones and mediators, such as human chorionic gonadotropin (β-hCG), human placental lactogen (hPL), placental growth hormone (PGH), progesterone, estrogen, leptin, and corticotropin-releasing hormone (Evain-Brion and Malassine, 2003; Costa, 2016). Furthermore, growth factors and cytokines, summarized as endocrine-active molecules, like vascular endothelial growth factor (VEGF), placental growth factor (PIGF), and transforming growth factor β (TGF-β), are synthesized and released by the STB (Costa, 2016).

Once platelets reach the intervillous space through the spiral arteries, they are exposed to an epithelial-like surface rich in pro- and anticoagulant factors, either membrane-bound or soluble, and extracellular components, like fibrin deposits (Kaufmann *et al.*, 1996; Lanir *et al.*, 2003). Platelet adhesion to placental villi is a common phenomenon from 5 weeks of gestation onwards and may lead to increased release of platelet-derived factors (Forstner *et al.*, 2020). According to Burnouf *et al.* (2016), platelet-derived factors include adhesion molecules, immunological mediators (e.g. complement factors), regulators of growth and angiogenesis, and coagulation factors, and may contribute to an inflammatory microenvironment in the intervillous space. These platelet-derived inflammatory chemokines and cytokines are not only involved in the generation of perivillous fibrinoid but can also hamper the synthesis of β-hCG of the STB, the major hormone to sustain pregnancy in the first trimester (Kaufmann *et al.*, 1996; Forstner *et al.*, 2020; Chen *et al.*, 2025). HCG mainly sustains progesterone production by the corpus luteal cells in the ovaries during the first 2 months of pregnancy, regulating fetal and placental growth and protecting pregnancy from immune rejection (Cole, 2010). Studies have shown that a high total hCG concentration in maternal blood in early pregnancy is associated with an up to 2.7-fold increased risk for preeclampsia (Barjaktarovic *et al.*, 2019).

Platelets are increasingly recognized as important contributors to the formation of perivillous fibrinoid at the maternal–fetal interface. Fibrin deposits can be detected already at very early stages of gestation; however, in the third trimester, enhanced platelet adhesion and activation are associated with increased perivillous fibrin deposition (Cheloufi *et al.*, 2024). Excessive accumulation of fibrin at the placental surface has been linked to placental insufficiency and is a recurrent phenomenon in pregnancies complicated with preeclampsia or fetal growth restriction (FGR) (Dusse *et al.*, 2011). However, it is still unclear whether platelet activation and the local release of platelet-derived factors initiate damage to the STB, thereby promoting secondary fibrin deposition, or whether primary STB lesions expose extracellular matrix components that subsequently provide a substrate for platelet adhesion and aggregation (Forstner *et al.*, 2021). Important players of the fibrinolytic system are also the plasminogen activator inhibitors type 1 (PAI-1) and type 2 (PAI-2). Besides their expression in the syncytiotrophoblast and extravillous trophoblasts and their role in inhibiting extracellular matrix (ECM) degradation, increased concentrations of PAI-1 in maternal blood are associated with recurrent pregnancy loss, preeclampsia, FGR, and endometriosis (Ye *et al.*, 2017; Nonn *et al.*, 2025). Given that the fibrinolytic system is suppressed during pregnancy, contributing to a procoagulant environment, studies have shown that pregnancies complicated with preeclampsia have an even higher shift towards a procoagulant environment compared to healthy controls (Hale *et al.*, 2012). Interestingly, even though preeclamptic patients develop an increased procoagulant environment, their platelet count is significantly lower even before the onset of preeclampsia and in the second trimester of pregnancy (Woldeamanuel *et al.*, 2023). Also, the mean platelet volume was shown to be significantly higher in women with severe preeclampsia compared to controls, which indirectly reflects increased platelet activation and aggregation (Chu *et al.*, 2010; Bellos *et al.*, 2018).

## Platelet–trophoblast crosstalk shapes placental function

The intervillous space provides a combination of coagulation factors, either soluble or membrane-bound, pregnancy-specific hormones, trophoblast-derived factors, and EVs, which may ‘educate’ platelets to support the hemostatic needs during pregnancy.

The villous trophoblast surface area increases from about 0.3 m^2^ at the end of the first trimester to 5 m^2^ during the late second trimester to a total expansion of about 12 m^2^ at term (Benirschke and Kaufmann, 2000; Wang, 2010; Masserdotti *et al.*, 2024). While the total placental volume can be estimated to be about 500–800 ml at term, the volume of the intervillous space (IVS) accounts roughly for 30–40%, resulting in an IVS volume of about 180–250 ml in a term placenta (Mayhew, 2009). Interestingly, it has been calculated that the entire IVS maternal blood pool is exchanged about 2–3 times per minute (Wang, 2010). Related to the maternal platelet count, an estimated number of about 10–15 × 10^10^ platelets pass through the IVS each minute in a steady state. As these numbers suggest, there would be a lot of possibilities for maternal platelets to be in contact and have interactions with fetal cells, especially the syncytiotrophoblast.

The interaction between syncytiotrophoblast and platelets is tightly regulated, as studies have shown that platelets can alter the expression pattern of STB surface proteins. Receptors, expressed at the surface of the STB, such as the G-protein-coupled sphingosine-1-phosphate receptors (S1PR1–5), are regulated by platelet-derived factors. A recent study has shown a downregulation of S1PR2 in trophoblast cells exposed to platelet-derived factors (Lyssy *et al.*, 2023). This indicates that platelets are not only involved in coagulation but also in the regulation of trophoblast signaling pathways linked to inflammatory responses and the regulation of vascular function (Lyssy *et al.*, 2023).

**Figure 3.**
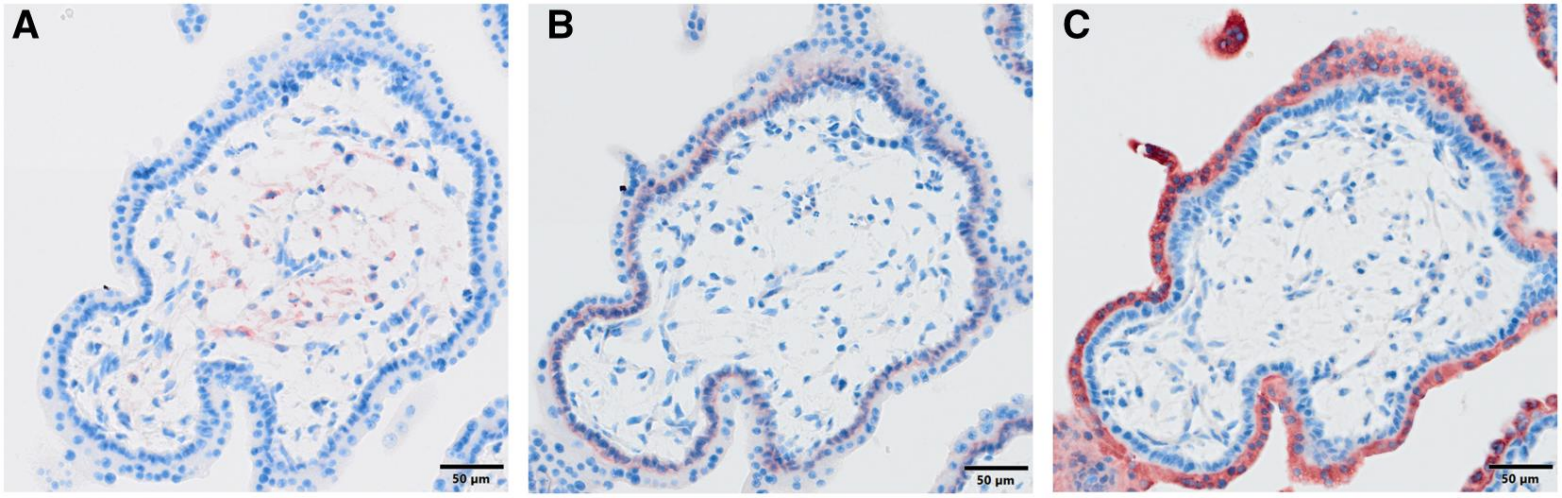
**Localization of pro- and anti-coagulant factors on placental villi**. First trimester placental villi were stained for tissue factor (TF) (**A**), tissue factor pathway inhibitor (TFPI)-1 (**B**) and TFPI-2 (**C**) as previously described (Lyssy *et al*., 2025a). TF, a strong pro-coagulant factor, could be located within the mesenchyme of fetal villi (A). TFPI-1 seemed to be expressed weekly in the villous cytotrophoblast (B), while TFPI-2 was located exclusively in the syncytiotrophoblast layer. Scale bars represent 100 µm.

The STB expresses and secretes certain pro- and anticoagulant factors to sustain balanced hemodynamics within the intervillous space. A major initiator of the extrinsic blood coagulation pathway is tissue factor (TF) and its inhibitors, tissue factor pathway inhibitor (TFPI)-1 and TFPI-2. TF, a cell surface glycoprotein involved in the coagulation cascade, is normally not expressed by cells exposed to blood flow. Only TFPI-2, which weakly inhibits thrombin and factor Xa and therefore serves as an anticoagulant factor in the human placenta, is predominantly expressed by the STB (Fig. 3) (Faulk *et al.*, 1990; Iochmann *et al.*, 2002; Lanir *et al.*, 2003; Mayhew, 2009; Kobayashi *et al.*, 2023).

Von Willebrand factor (vWF), as a procoagulant factor, synthesized by endothelial cells, plays an important role in hemostasis by mediating platelet adhesion to the subendothelial surface and promoting platelet aggregation (Parra-Cordero *et al.*, 2011). VWF has also been detected in the STB, raising the possibility that trophoblast damage may expose subepithelial vWF and thereby facilitate platelet adhesion to the placental villi, analogous to the mechanism by which platelets bind to vWF following subendothelial exposure during vascular injury (Parra-Cordero *et al.*, 2011). Several studies have shown that plasma levels of vWF are increased in preeclamptic pregnancies compared to controls (Nadar *et al.*, 2004; Hulstein *et al.*, 2006; Peng *et al.*, 2010). In regard to anticoagulant mechanisms of the placenta, thrombomodulin (Turner *et al.*, 2016), nitric oxide (Conrad *et al.*, 1993; Sanyal *et al.*, 2000), and endothelial protein C receptor (Faioni *et al.*, 2015) expressed at the syncytiotrophoblast surface, ensure a balanced hemostasis in the intervillous space (Valdes *et al.*, 2009; Kohli *et al.*, 2021).

Furthermore, the trophoblast is equipped with ectonucleotidases, which convert extracellular ATP, released in response to inflammation, into an anti-thrombotic adenosine-rich environment. The placental ectonucleotidases CD39 and CD73, expressed at the apical side of the syncytiotrophoblast, have been shown to be significantly deregulated in pregnancies complicated by preeclampsia, suggesting an increased demand for extracellular ATP hydrolysis. This mechanism may present an important anti-coagulant mechanism of the placenta in this syndrome (Table 2) (Forstner *et al.*, 2023).

**Table 2.** Placenta-derived pro- and anticoagulant factors and their interactions with platelets.

Placental factor	Primary placental source	Interaction with platelets
**Procoagulant factors**		
TF	Decidual fibrin deposits; uterine epithelium; mesenchyme of the placenta (Boer *et al.*, 2007; Ayala-Ramírez *et al.*, 2021) (Figure 3)	Indirect platelet activation via activation of FX and thrombin generation (Zelaya *et al.*, 2018)
Fibrin	Perivillous fibrinoid (Kaufmann *et al.*, 1996)	Binding to platelet glycoprotein VI (GPVI) receptor (Alshehri *et al.*, 2015)
PAI-1 and -2	STB, EVT (Ye *et al.*, 2017)	Stabilizes fibrin by inhibition of the fibrinolytic system, supporting platelet adhesion (Hvas and Larsen, 2023)
vWF	Placental endothelium, STB (Parra-Cordero *et al.*, 2011)	Promotes platelet adhesion via binding to platelet receptors glycoprotein (GP) Ib-IX-V and αIIbβ3 integrin (Bryckaert *et al.*, 2015)
**Anticoagulant factors**		
NO	STB, placental endothelium (Conrad *et al.*, 1993; Krause *et al.*, 2011)	Inhibits platelet activation via increasing cGMP and a subsequent reduction of cytosolic calcium (Freedman *et al.*, 1997)
Ectonucleotidases	STB (Forstner *et al.*, 2023)	Degradation of ATP/ADP and reduction of ADP-mediated platelet stimulation (Chaurasia *et al.*, 2020)
TM	Placental endothelium, STB (Stortoni *et al.*, 2010; Turner *et al.*, 2016)	Converts thrombin into an anticoagulant; inhibitor of thrombin-mediated platelet activation (Brummel-Ziedins and Mann, 2018)
EPCR	Placental endothelium, STB (Faioni *et al.*, 2015; Andres *et al.*, 2022)	Facilitates activation of protein C by the thrombin-TM complex and limits thrombin generation (Castillo *et al.*, 2020)
TFPI-1	STB, vascular endothelium, EVT (Edstrom *et al.*, 2000)	Indirect inhibition of platelet activation via inhibition of TF and FVIIa by binding to FXa (Edstrom *et al.*, 2000)
TFPI-2	Highly expressed in STB (Lanir *et al.*, 2003)	Indirect inhibition of platelet activation via strong inhibitor of FXa (Lanir *et al.*, 2003)

EPCR, endothelial protein C receptor; EVT, extravillous trophoblast; FVIIa/FX/FXa, factor VIIa/X/Xa; NO, nitric oxide; PAI, plasminogen activator inhibitor; STB, syncytiotrophoblast; TF, tissue factor; TFPI, tissue factor pathway inhibitor; TM, thrombomodulin; vWF, von Willebrand factor.

Other important mediators of communication between the fetal placenta and the maternal circulation, which cannot be neglected, are EVs (Kupper and Huppertz, 2022). Syncytiotrophoblast-derived EVs (STB-EVs) are known carriers of biologically active molecules that can traffic to local or distant targets and execute defined biological functions (Margolis and Sadovsky, 2019). STB-EVs have been shown to quickly associate with maternal platelets and can even be internalized by them (Tannetta *et al.*, 2015).

Although there is hardly any evidence in healthy pregnancies, studies have reported that in preeclamptic pregnancies, STB-EVs can promote platelet activation and priming through multiple mechanisms, including the presentation of tissue factor and phosphatidylserine, which enhance thrombin generation and subsequent activation of protease-activated receptors (Tannetta *et al.*, 2017). STB-EVs from preeclamptic patients are proinflammatory, anti-angiogenic, and procoagulant, which promote different facets of endothelial dysfunction. It has therefore even been discussed whether the analysis of circulating STB-EVs in maternal peripheral blood could be used as placental liquid biopsies in preeclamptic patients (Awoyemi *et al.*, 2022).

However, platelets can also release EVs (P-EVs), which are usually characterized by their rich molecular cargo inherited from their original parent megakaryocytes. Previous studies have reported that P-EV counts are stable throughout gestation (Buca *et al.*, 2023), which is an interesting observation given the fact that platelet count gradually decreases with ongoing pregnancy. However, compared to non-pregnant women, there is still an increase in circulating P-EV count, possibly due to the physiologic hypercoagulable state during pregnancy (Buca *et al.*, 2022). The procoagulant surface of P-EVs, characterized by phosphatidylserine exposure, further promotes thrombin generation, which can potentially influence trophoblast behavior via protease-activated receptor signaling (Tripisciano *et al.*, 2017).

In pathological conditions such as preeclampsia, isolated P-EVs differ phenotypically and functionally from those of healthy pregnant controls. Previous studies have discussed the possibilities of EVs having a pathogenic function in preeclampsia, in more detail, that EVs can trigger accumulation of activated platelets specifically in the placenta, promoting inflammasome activation within trophoblast via purinergic signaling, which is required and sufficient for a preeclampsia-like phenotyping in pregnant mice (Kohli *et al.*, 2016). Moreover, thrombo-inflammatory pathways, induced by procoagulant EVs, can modulate trophoblast morphology and differentiation, suggesting a crucial role during the development of pregnancy complications as well as possible therapeutic targets (Markmeyer *et al.*, 2021).

## Pregnancy-related hormones affect platelet behavior

Besides pro- and anticoagulant factors, which are either expressed at the STB membrane or secreted into the intervillous space, platelet activation might also be influenced by the hormone cocktail present at any given time of gestation (Table 3). Although most studies have neglected the particular hormone status during pregnancy, several findings predict a sex-dependent difference in platelet reactivity due to differences in hormonal status (Becker *et al.*, 2006; Koupenova *et al.*, 2015).

**Table 3.** Platelet-hormone interaction in the intervillous space during the first trimester of gestation.

Hormone	Side of interaction on platelets	Influence on platelet activity
Estrogen	Estrogen receptor β (ER β) (Dupuis *et al.*, 2019)	Contradictory results on platelet activation (Dupuis *et al.*, 2019)
Progesterone	No known primary progesterone receptor; systemic or indirect interaction (Blackmore, 2008)	Dampens platelet aggregation, modulates intracellular Ca^2+^ signaling (Blackmore, 2008)
Placental growth factor	Expression of VEGFR (Flt1), binds PIGF (Selheim *et al.*, 2002)	Sequester PIGF, release it upon activation (Selheim *et al.*, 2002)
Leptin	Functional leptin receptor (Giandomenico *et al.*, 2005)	Leptin potentiates ADP-induced platelet aggregation and stimulates platelet adhesion (Elbatarny and Maurice, 2005)

PIGF, placental growth factor; VEGFR: vascular endothelial growth factor receptor.

The interaction between platelets and progesterone, for example, is mainly based on indirect or systemic mechanisms, since platelets are anucleated cells, which therefore lack the classical nuclear steroid hormone receptor, progesterone receptor-A, -B, or -C (Azeez *et al.*, 2021). A study has shown that progesterone alone did not affect Ca^2+^ influx in platelets, but their metabolites, such as pregnanolone, which in turn can cause rapid signaling responses in platelets by increasing intracellular calcium, leading to platelet activation (Blackmore, 2008).

Platelets also possess a functional VEGF-related receptor, fms-like tyrosine kinase-1 (Flt1), and a kinase-insert domain region, also known as KDR, to interact with VEGF and PIGF (only Flt1) during pregnancy. VEGF has been described to potentiate the effect on platelet activation, both through its autocrine platelet behavior and through VEGF–endothelial interactions (Selheim *et al.*, 2002).

Another strong correlation between placenta-derived hormones and platelets was shown by Giandomenico *et al.* (2005), who found a potentiating effect of leptin on platelet aggregation, induced by low concentrations of ADP, collagen, and epinephrine. Interestingly, this effect was only found in 40% of all donors, referred to as responders, whereas 60% of all donors were non-responders, which was explained by higher expression levels of the signaling form of the leptin receptor in platelets from responders. Both maternal circulating leptin as well as placental leptin expression are elevated in pregnancies accompanied by preeclampsia, contributing to hyperactive inflammation and oxidative stress, but possibly also an amplified hypercoagulation (Zeng *et al.*, 2023).

The interaction between estrogen and platelets is pointed out in a study, which suggests periodic fluctuations in platelet adhesion to collagen correlating with peak levels of the estrogen E2 concentration in maternal plasma (Tarantino *et al.*, 1994; Dupuis *et al.*, 2019). A study of Rosenblum *et al.* (1985) showed an enhancement of platelet aggregation in mice when treated with 0.5 mg estradiol, while another study from Valéra *et al.* (2012) reported a marked decrease in platelet responsiveness in mice exposed to high physiological levels of estradiol. However, contradictory results are reported and thus may be explained by the use of different estrogen concentrations and different experimental designs (Dupuis *et al.*, 2019).

Platelets present several surface receptors, including but not limited to proteinase-activated receptors (PARs), glycoproteins, and integrins, which are responsible for the communication with other cell types, such as neutrophils or monocytes (Forstner *et al.*, 2021; Kohli and Blois, 2025). The glycoprotein IIb/IIIa (GPIIb/IIIa) is one of the most abundant receptors on platelets. Upon platelet activation, its ligand affinity increases, and vWF, fibrinogen, and prothrombin are able to bind (Hashemzadeh *et al.*, 2022). While the influx of Ca^2+^ from the extracellular environment leads to the activation of the receptor, production of cAMP and cGMP by nitric oxide (NO) or prostacyclin (PGI_2_) leads to an inhibition of the platelet receptor GPIIb/IIIa. Besides its effect on platelet adhesion and aggregation, estrogen has been shown to suppress GPIIb/IIIa activation by increasing cAMP, despite unchanged receptor expression (Nakano *et al.*, 2002; Hashemzadeh *et al.*, 2022). The influence of pregnancy-specific hormones on the activation status of this receptor might subsequently also influence the binding of platelets to immune cells. As neutrophils and monocytes are already known to interact with platelets via P-selectin or CD40L, especially with GPIIb/IIIa, the activation status of this receptor might be of high importance when it comes to platelet–immune cell interactions (Rayes *et al.*, 2020). This tempts to speculate that pregnancy-associated hormones may indirectly modulate platelet interactions with immune cells via altering receptor expression or activation status.

## Placenta-exposed platelets re-enter the maternal systemic circulation

A key question, when elucidating the journey of platelets through the pregnant maternal circulation, is to what extent platelets are consumed, retrained, or destroyed within the intervillous space. The decline in platelet counts toward the end of pregnancy is often explained as a combination of hemodilution and localized consumption within the utero-placental circulation (Reese *et al.*, 2019).

A study has shown that a minority of platelets entering the intervillous space are permanently consumed, primarily through adhesion to the STB or perivillous fibrinoid or undergoing activation and subsequent degranulation (Forstner *et al.*, 2020). Although those mechanisms significantly influence trophoblast physiology, the majority of platelets will transit through the intervillous space without irreversible activation or adhesion.

Once platelets have passed the intervillous space, potentially primed with placenta-derived pro- and anticoagulation factors or internalized EVs, they re-enter the maternal vasculature via the uterine veins before entering the common iliac veins and the inferior vena cava and finally reaching the right atrium of the heart (Benninghoff, 2004). Unlike the arterial inflow through the spiral arteries, veins are very susceptible to external forces, which is why parameters such as intra-abdominal pressure should be taken into account when it comes to facilitate an efficient drainage of the large blood volume within the intervillous space (Gyselaers and Dreesen, 2025). Platelets that have not adhered or undergone irreversible activation are carried back into the systemic circulation with maternal blood, thereby completing the circulatory loop. Platelets can further participate in systemic hemostasis, regardless of whether they were placenta-primed or not.

The average lifespan of a platelet in humans is about 10 days (Lebois and Josefsson, 2016). During this time, a red blood cell, circulating in the same bloodstream, can traverse capillaries between 1000 and 2000 times per day, which equals roughly one full transit per minute (Rogers and Lew, 2021). Consequently, it can be hypothesized that, without prior consumption, a platelet has the potential to theoretically pass through the human body, including the placental vasculature, approximately 14.4 × 10^3^ times during its lifespan.

Taken together, the journey of platelets through the placenta can be conceptualized as a regulated transit through a highly specialized vascular compartment, rather than as a site of indiscriminate platelet trapping (Greer *et al.*, 2014). The balance between platelet activation, adhesion, and secretion is tightly controlled and appears essential for placental homeostasis. However, once this system is imbalanced, hemostatic complications during pregnancy occur and may eventually lead to thrombotic events.

## Conclusion

Pregnancy requires profound adaptations in maternal hemodynamics and platelet biology. Starting with megakaryopoiesis, the first hallmarks of pregnancy adaptation are measurable with an increased platelet turnover. At the same time, the total number of platelets is steadily decreasing toward the end of pregnancy. Once in the maternal circulation, platelets are exposed to a mix of pregnancy-specific mediators, mainly trophoblast-derived, and may get primed by this mix of chemokines, cytokines, hormones, and STB-EVs. Besides a shift in the soluble factors toward a procoagulant status during gestation, the journey further leads them through the uterine arteries into the utero-placental blood flow, where enhanced priming by high concentrations of trophoblast-derived factors might take place. Platelets in the placental intervillous space may either get activated, adhere to the STB, or re-enter the maternal vasculature without being activated or sequestrated within the intervillous space.

Although preeclampsia is a ‘syndrome of hypotheses,’ with not a single hypothesis explaining the etiology of the full spectrum of the syndrome, changes in (placental) hemodynamics and the behavior of platelets might contribute to the development of this syndrome (Gyselaers and Dreesen, 2025; Huppertz, 2026c).

However, major research gaps remain and point to the need for quantitative data on platelet flow through the intervillous space. There is still a lack of appropriate *in vitro* models, including advanced placental perfusion models, imaging of platelet dynamics, and the integration of clinical hematological data with placental pathology. Although preeclampsia is not primarily a coagulation disorder but rather a systemic syndrome associated with placental dysfunction and systemic vascular and hemostatic consequences, it is important to address research gaps relating to hemodynamics and uterine blood flow in the fight against this pathology. Understanding these mechanisms is essential for the early detection of preeclampsia, risk stratification, and the development of future therapeutic approaches.

## Data Availability

All data are incorporated into the article.
